# Retraction Note: Targeted suicide gene therapy for glioma using human embryonic stem cell-derived neural stem cells genetically modified by baculoviral vectors

**DOI:** 10.1038/s41434-020-0135-y

**Published:** 2020-02-28

**Authors:** Y. Zhao, D. H. Lam, J. Yang, J. Lin, C. K. Tham, W. H. Ng, S. Wang

**Affiliations:** 1 Drug and Gene Delivery, Institute of Bioengineering and Nanotechnology, Singapore, Singapore; 2 Department of Biological Sciences, National University of Singapore, Singapore, Singapore; 3 Department of Medical Oncology, National Cancer Centre Singapore, Singapore, Singapore; 4 Department of Neurosurgery, National Neuroscience Institute, Singapore, Singapore

**Retraction Note to: Gene Therapy**

10.1038/gt.2011.82.

The editor has retracted this article [1] following an investigation by Agency for Science, Technology and Research (A*STAR), Institute of Bioengineering and Nanotechnology, Singapore, which found evidence of fabricated animal survival data presented in Fig. 7C in the article to make the results statistically significant. DHL, JY, JL, CKT, WHN, and SW agree to this retraction. YZ has agreed to this retraction but not to the wording of this retraction notice.
